# Integrated analysis of cell-in-cell related genes and immune microenvironment in heart failure

**DOI:** 10.3389/fcell.2026.1806426

**Published:** 2026-05-08

**Authors:** Linna Zhao, Yuepeng Zhou, Jiahuan Sun, Shupeng Liu, Weizhe Liu, Aiying Li

**Affiliations:** 1 Department of Biochemistry and Molecular Biology, College of Pharmacy, Hebei University of Chinese Medicine, Shijiazhuang, Hebei, China; 2 Department of Epidemic Febrile Disease, College of Traditional Chinese Medicine, Hebei University of Chinese Medicine, Shijiazhuang, Hebe, China; 3 Hebei Key Laboratory of Chinese Medicine Research on Cardio-Cerebrovascular disease, Shijiazhuang, Hebei, China

**Keywords:** biomarker, cell-in-cell, heart failure, immune microenvironment, single-cell transcriptomics

## Abstract

**Background:**

Heart failure (HF) is a major global public health challenge, and its pathogenesis involves the regulation of a complex immune microenvironment (IME). Cell-in-cell (CIC), as a non-classical form of cell-cell interaction, has been extensively studied in fields like oncology, but its role in HF remains unclear. This study aimed to systematically analyze the expression patterns and functions of CIC-related genes (CRGs) within the HF immune microenvironment.

**Methods:**

Based on transcriptomic data from public databases, CIC-related differentially expressed genes (DEGs) between HF and healthy samples were identified. Three machine learning algorithms—Random Forest, LASSO, and SVM-RFE—were employed to screen diagnostic markers and construct a nomogram model. Consensus clustering analysis was used to stratify HF patients into distinct subtypes based on CRG expression, and their immune infiltration characteristics were compared. Single-cell transcriptomic data were utilized to validate the cellular localization of key genes within the HF microenvironment. Experimental validation of key CRGs was performed using a transverse aortic constriction (TAC)-induced HF rat model.

**Results:**

A total of 21 CIC-related DEGs were identified. A diagnostic model comprising 10 core genes demonstrated high predictive performance in both the training and validation sets. Based on CRG expression, HF patients were classified into two subtypes: Subtype A was enriched with regulatory T cells and M2 macrophages, exhibiting an immunosuppressive and fibrotic phenotype; Subtype B was dominated by cytotoxic T cells and NK cell infiltration, displaying an immune-activated phenotype. Single-cell analysis revealed high expression of CTSK in fibroblasts and enrichment of GZMB in T/NK cells. Animal experiments confirmed the upregulation of LPAR2 and GZMB and the downregulation of IL-10 in the TAC model.

**Conclusion:**

CIC-related genes possess significant diagnostic value in HF and can distinguish HF subtypes with distinct immune microenvironment features. CRGs may participate in HF progression by regulating immune cell infiltration and fibrotic processes, providing a new perspective for understanding HF heterogeneity and developing targeted immunotherapies.

## Introduction

Heart failure (HF), characterized by the progressive decline of cardiac pumping function, represents the common end-stage of various cardiovascular diseases and constitutes a formidable global public health challenge. The World Health Organization estimates that over 64 million individuals worldwide are affected by HF, with a five-year mortality rate comparable to many malignancies and high rates of rehospitalization, imposing a substantial economic and social burden on healthcare systems (Gergely et al., 2024; Katoh et al., 2024; Li et al., 2024). Despite significant improvements in clinical outcomes for some patients with novel therapeutics such as angiotensin receptor-neprilysin inhibitors and sodium-glucose cotransporter two inhibitors, the overall prognosis remains suboptimal (Jaarsma et al., 2021; Rakisheva et al., 2025). This underscores that HF is far more than a simple disorder of myocardial contractility; it is a complex syndrome involving intricate interactions among diverse cell types, molecular pathways, and systemic processes (Rakisheva et al., 2025). Consequently, moving beyond the traditional hemodynamic framework to dissect its pathogenesis through deeper molecular and cellular interaction networks is pivotal for identifying novel therapeutic targets and biomarkers (Markousis-Mavrogenis et al., 2024).

Within this context, the central role of the immune system in HF pathogenesis has gained increasing recognition. Research indicates that a state of chronic low-grade inflammation persists both locally within the myocardium and systemically in patients with HF, regardless of ejection fraction status (HFrEF or HFpEF) (Paulus and Zile, 2021; Schiattarella et al., 2022). Immune cells, including macrophages, T cells, and neutrophils, infiltrate the cardiac tissue through complex networks, releasing a plethora of inflammatory cytokines and growth factors that directly drive cardiomyocyte hypertrophy, apoptosis, interstitial fibrosis, and microvascular dysfunction (Fragasso et al., 2025; Halade and Lee, 2022; Tang et al., 2022). For instance, neutrophil extracellular trap formation exacerbates cardiac dysfunction in dilated cardiomyopathy (Ichimura et al., 2024); imbalance in macrophage polarization (e.g., increased M2 phenotype) is closely associated with fibroblast activation and fibrosis (Wang Z. et al., 2023); and disturbances in T cell subsets, particularly regulatory T cells, affect the balance between myocardial repair and chronic inflammation (Lu et al., 2021). These collective findings establish the cardiac immune microenvironment as a decisive factor influencing HF progression and prognosis, positioning immunomodulation as a highly promising novel therapeutic direction (Andreadou et al., 2025; Markousis-Mavrogenis et al., 2024).

Although the centrality of immune inflammation is well-established, the specific cytological mechanisms governing the dynamic equilibrium of this complex microenvironment remain largely unexplored. In recent years, a non-classical cell biological phenomenon known as “cell-in-cell” (CIC) has garnered significant attention in fields such as oncology (Andreadou et al., 2025; Fais and Overholtzer, 2018). CIC refers to the process where one living cell is completely internalized by another living cell of either the same or different type, encompassing forms such as entosis and emperipolesis (Cunin et al., 2019). It serves not only as a mechanism for cell competition and the clearance of damaged cells but also plays specialized roles in immune surveillance, antigen presentation, and intercellular communication (Mihlan et al., 2024). For example, neutrophils can be internalized by megakaryocytes via emperipolesis, facilitating the transfer of membrane components (Cunin et al., 2019); in contexts of infection or inflammation, CIC may act as a unique conduit for propagating inflammatory signals (Mihlan et al., 2024). This process is tightly regulated by a specific set of genes (e.g., RhoA for cytoskeletal regulation, E-cadherin for cell adhesion, LC3 involved in autophagy) (Su et al., 2022; Wang et al., 2020). Intriguingly, many known CIC-related genes (CRGs) are also expressed during immune cell activation and cardiomyocyte stress responses (Humeres et al., 2022; Li et al., 2024). However, in stark contrast to the rich knowledge established in cancer research (Liu and Yang, 2023; Song et al., 2023; Wang R. et al., 2023), CIC and its related gene networks remain virtually unexplored in HF, particularly regarding their interplay with the cardiac immune microenvironment. Examining immune differences between samples from healthy individuals and HF patients, as well as among different HF subtypes, and studying how CRGs shift in response to these changes, may deepen our comprehension of HF mechanisms from a fresh perspective.

This study comprehensively assesses the regulatory patterns of CRGs in the context of HF. Our findings indicate that CRGs can effectively differentiate between samples from healthy individuals and HF patients and their expression patterns were validated in rats with HF induced by transverse aortic constriction (TAC). Furthermore, notable correlations were observed between the abundance of infiltrating immune cells and CRGs, implying a strong association between CRGs and immune regulatory processes. Samples from HF individuals were categorized into two distinct CIC patterns based on the expression patterns of ten CRGs. Notably, immune features were observed within these subtypes and the biological functions specific to each subtype were compared. Additionally, the expression of the ten CRGs in the HF microenvironment was analyzed using the HF single-cell dataset. These collective findings underscore the substantial influence of CRGs on the IME of HF.

## Materials and methods

### Data preprocess

All data used in this study are from public datasets. We downloaded two bulk gene expression datasets GSE141910 (200 HF, 166 Healthy) (Flam et al., 2022), GSE57338 (177 HF, 136 Healthy) (Liu et al., 2015) from Gene Expression Omnibus database (http://www.ncbi.nlm.nih.gov/geo/). GSE141910 was based on the GPL16791 platform Illumina HiSeq 2,500 (*Homo sapiens*), while the GSE57338 was based on the GPL11532 platform [HuGene-1_1-st] Affymetrix Human Gene 1.1 ST Array [transcript (gene) version]. All data were preprocessed and obtained by R package “GEOquery (Davis and Meltzer, 2007). To mitigate platform-specific batch effects, differential expression analysis was conducted exclusively on GSE141910, while GSE57338 served strictly as an independent validation cohort. Consensus clustering analysis was also performed solely on GSE141910 to avoid batch effects from cross-dataset integration. Gene probes were annotated as gene symbols. Probes without matching gene symbols and matching multiple symbols were excluded. Gene expression value of duplicate gene symbol was calculated as the max value. 101 CIC-related genes were downloaded from previous literature (Zhang et al., 2024) and are provided in Supplementary Table S1. We also downloaded single-cell sequencing data containing 5 Transmural LV Apex samples of HF from GSE183852 (Koenig et al., 2022). The data were analyzed according to the post-quality control given and the cells were according to the data in the original study.

### Differentially expressed gene (DEG) analysis and functional analysis

R package limma (Ritchie et al., 2015) were used to identify CIC-related genes (CRGs) between HF cases and Healthy cases in GSE141910. Importantly, the cut-off value was set as FDR (false discovery rate) < 0.05 and log2 |fold change (FC)| > 0.5. The Metascape (Zhou Y. et al., 2019) database was used for the subsequent GO and KEGG enrichment analyses on these CIC-related DEGs, and an adjusted P value <0.05 was considered statistically significant.

### Screening and validation of CIC diagnostic markers

New and important biomarkers for HF were screened using three machine-learning algorithms: random forests (RF), least absolute shrinkage and selection operator (LASSO) logistic regression, and support vector machine-recursive feature elimination (SVM-RFE). The “randomForest” (Wang Y. et al., 2023) R package in R was used to implement the random forest technique in this study. This study carried out LASSO logistic regression investigation with the R package “glmnet” (Engebretsen and Bohlin, 2019), and minimal lambda was considered optimal. In our study, the selection of optimization parameters was cross-verified by a factor of 10, and the partial likelihood deviation met the minimum criteria. The genes that have traits in common of the three classification models discussed before were then selected for additional nomogram model. The validation set for the complete analysis of the usefulness of significant biomarkers will be the datasets from GSE57338. It was evaluated based on the study of receiver operating characteristic (ROC) curves, and the area under the curve (AUC) was calculated to measure the predictive capability of the nomogram model. Then decision curve analysis was drawn to assess the predictive accuracy of the nomogram.

### Identification of CIC modification pattern

We performed an unsupervised cluster analysis of the 101 CIC related genes expressions using the “ConsensusClusterPlus” (Wilkerson and Hayes, 2010) package to identify different CIC related clusters. The “K-Means” algorithm was applied and “euclidean” was used as a measure of distance, accompanied by resampling of 80% of the items and 1,000 replications. The optimal k value was determined according to the proportion of ambiguous clustering (PAC). PCA was conducted to further validate the CIC related genes expression patterns in different modification patterns. The infiltrating immunocyte abundance score, immune checkpoint and HLA gene expression among the two distinct modification patterns were compared by the Wilcox test. Gene set enrichment analysis (GSEA) was performed to determine the key pathways and core genes between distinct CIC modification patterns. He enriched pathways were arranged in the order of their normalized enrichment scores, and those with P < 0.05 were chosen for further analysis.

### Single-cell RNA-seq (scRNA-seq) data analysis

scRNA-seq data was obtained from GSE183852 and was analyzed with Seurat (https://github.com/satijalab/seurat) (Slovin et al., 2021). Cells with <300 or >5,000 genes and mitochondrial gene fragments>10% were filtered. The remaining cells were merged into one gene expression count matrix, and the count data were normalized and scaled using Seurat’s functions of NormalizeData and ScaleData. Dimension reduction and clusters identification of cells were implemented by RunUMAP and Findclusters functions. After cluster classification, different cell clusters were identified and annotated by SingleR (Zheng et al., 2023) R package. The’featureplot’ function is used to show the expression of genes.

### CeRNA-network construction

Transcription factors (TFs) are proteins that can bind to specific DNA sequences and regulate the expression of genes. NetworkAnalyst 3.0 (https://www.networkanalyst.ca/) (Zhou G. et al., 2019) was used to analyze the interaction of the common genes and transcription factors. MicroRNAs (miRNAs), which mediate target mRNA degradation or translation inhibition, are one class of endogenous short non-coding RNAs. The common genes were submitted to NetworkAnalyst 3.0 to generate a common genes-miRNA coregulatory network.

### Establishment of the TAC animal model

The TAC animal model and normal tissue samples used in this study were sourced from the laboratory research group. Male Sprague-Dawley rats (150–170 g) were purchased from Beijing Charles River Laboratory Animal Technology Co., Ltd. and housed under standard conditions (23 °C, 55%–60% humidity) with free access to food and water for 1 week prior to the experiment. To establish the heart failure model, rats were anesthetized with an intraperitoneal injection of 0.3% pentobarbital sodium and subjected to TAC using a 27-gauge needle and a 6–0 silk suture to ligate the aorta, as previously described. The sham-operated group underwent an identical surgical procedure without aortic ligation. Four weeks post-surgery, transthoracic echocardiography was performed to confirm the successful establishment of the HF model. Rats were then randomly divided into experimental groups (n = 6 per group) for subsequent analysis. This research group had previously published articles utilizing these tissue samples (Zhou et al., 2022). In this study, these tissue samples were further used for the field of HF research.

### Western blot analysis

Proteins were extracted from left ventricular myocardial tissues of rats using a lysis buffer consisting of RIPA buffer, phenylmethylsulfonyl fluoride, a protease inhibitor cocktail (from Roche, Switzerland), and phosphatase inhibitors (provided by Wuhan Servicebio, China). The protein content was quantified using a bicinchoninic acid kit, and proteins were subsequently separated through SDS-PAGE gel electrophoresis. Subsequently, the proteins were transferred onto a polyvinylidene fluoride membrane (from Millipore). The membrane was blocked with 5% nonfat dry milk in Tween/Tris-buffered saline at room temperature for 90 min. Following this, the membrane was incubated with primary antibodies overnight at 4 °C. The employed antibodies encompassed anti-NPPA (rabbit polyclonal, 1:2000, 27426-1-AP, Proteintech), anti-BNP (mouse polyclonal, 1:1,000, ab239510, Abcam), anti-beta MHC (rabbit polyclonal, 1:1,000, 22280-1-AP, Proteintech), anti-Collagen I (rabbit polyclonal, 1:1,000, 14695-1-AP, Proteintech), anti-Collagen III (rabbit polyclonal, 1:1,000, 22,734–1-AP, Proteintech), anti-α-SMA (rabbit polyclonal, 1:4,000, ab32575, Abcam), anti-IL-10 (rabbit polyclonal, 1:1,000, #HA722032, HUABIO), Anti-EDG4 (rabbit polyclonal, 1:500, CPA6684, Cohesion Biosciences), anti-Granzyme B (rabbit polyclonal, 1:1,000, 13588-1-AP, Proteintech) and anti-GAPDH (mouse monoclonal, 1:10,000, 60,004–1-Ig, Proteintech). The following day, the membrane was incubated with the secondary antibody at room temperature for 90 min. Immunoreactive bands were detected utilizing the ECL chemiluminescent substrate kit and visualized using the chemiluminescent imager OmegaLum W (Minneapolis, MN, United States).

### Real-time fluorescent quantitative PCR analysis

Total RNA was extracted from cardiac tissues and cardiac fibroblasts using the Total RNA Kit II (R6934-01, Omega, United States), and cDNA was synthesized by UnionScript First-strand cDNA Synthesis Mix for qPCR (with dsDNase). Subsequently, qRT-PCR analysis was performed using the QuantStudio 1 Real-Time PCR System (Thermo Fisher Scientific, United States), and the expression levels of IL-10, LPAR2 and GZMB were detected using GS AntiQ qPCR SYBR Green Fast Mix (Universal). GAPDH mRNA was used as the internal reference for normalization. All primer sequences were synthesized by Sangon Biotech (Shanghai, China) (Supplementary Table S2).

### Statistical analysis

All data calculations and statistical analysis were performed using R programming (https://www.r-project.org/, version 4.1.1). Differences between the two groups were analyzed by Wilcoxon tests (mean ± SD), and *P* < 0.05 indicated statistical significance (ns: no significance, *P < 0.05, **P < 0.01, ***P < 0.001, ****P < 0.0001). For correlation analysis, we calculated the Pearson correlation coefficient, as indicated. P values <0.05 were considered significant.

## Result

### Variant landscape of CIC l related genes in HF patients

**FIGURE 1:**
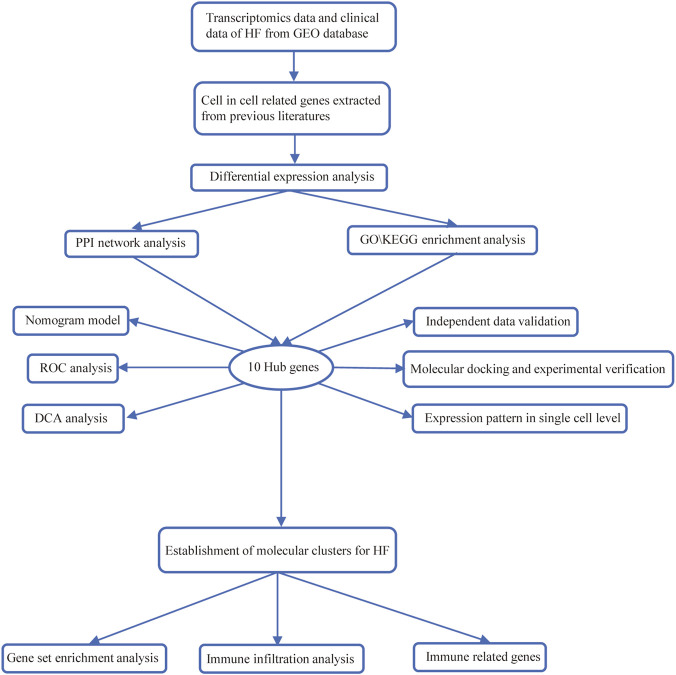
Schematic overview of the study workflow.

**FIGURE 2:**
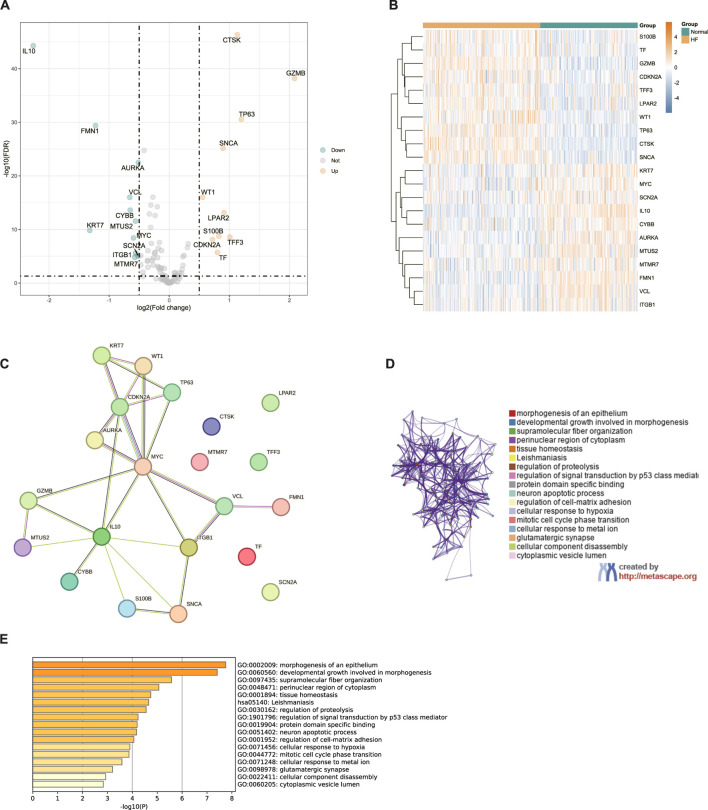
Variant Landscape of CIC related genes in HF **(A)**Volcano plot illustrating CIC related DEGs between HF and normal tissues (green: downregulated DEGs; yellow: upregulated DEGs; grey: unchanged genes). Points with labels represent significant DEGs with FDR <0.05 and |log2FC| > 0.5 **(B)** Heatmap of differential analysis between HF and normal group. Blue indicates the normal cohort, yellow denotes the HF cohort, blue squares represent low expression, and yellow squares signify high expression **(C)** PPI network of CIC related DEGs **(D,E)** GO term and KEGG enrichment analyses were applied to the CIC related DEGs.

The flow chart of this present study was demonstrated in Figure 1. We determined a total of 21 CIC-related DEGs between HF and normal samples from GSE141910 database which were showed in Figure 2A. The expression of each of the 21 CIC-related DEGs in the GSE141910 is shown in Figure 2B. Afterward, we constructed a protein–protein interaction network to elucidate the intricate relevance of CIC DEGs -associated proteins (Figure 2C). GO and KEGG enrichment analysis revealed that CIC-related DEGs were associated with morphogenesis of an epithelium, developmental growth involved in morphogenesis, supramolecular fiber organization, perinuclear region of cytoplasm, neuron apoptotic process, regulation of cell-matrix adhesion (Figures 2D,E).

### Construction of a nomogram based on CIC related genes

**FIGURE 3:**
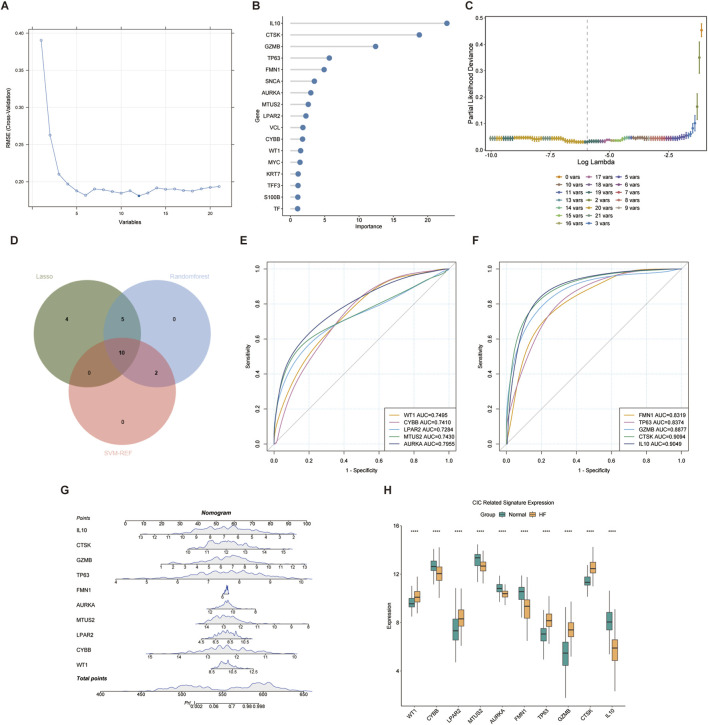
Selection of Signature Genes from CIC related DEGs using Machine Learning Algorithms **(A)** Significant features selected by the SVM-RFE algorithm **(B)** Optimal biomarkers screened by the RF algorithm **(C)** Variable selection in the LASSO model **(D)** Venn diagram showing the overlap of genes across three algorithms **(E,F)** ROC curves were utilized to evaluate the capability of diagnostic markers in distinguishing healthy from HF samples, with performance quantified by the AUC value **(G)** A nomogram was developed for HF prediction based on 10 genes **(H)** Boxplot illustrating the differential expression of CIC related features between HF tissues and normal tissues.

**FIGURE 4:**
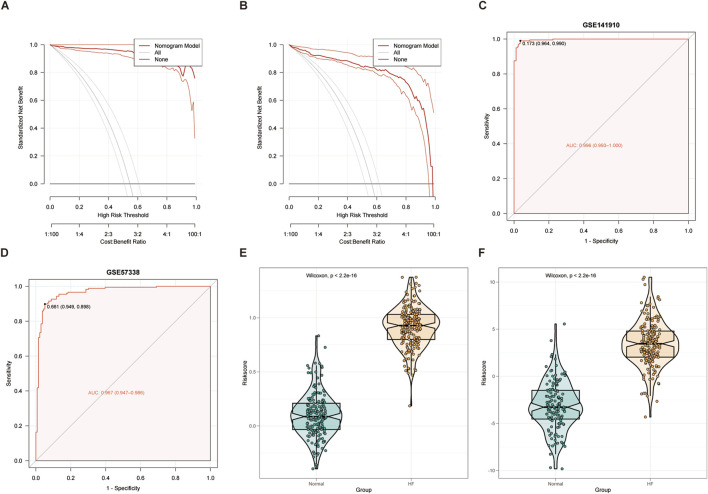
Validation analysis of nomogram model in HF. **(A,B)** Decision Curve Analysis was performed to evaluate the risk prediction nomogram for HF in the GSE141910 and GSE57338 datasets. **(C**,**D)** ROC curve analysis was conducted to validate the risk prediction nomogram for HF using data from GSE141910 and GSE57338. **(E**,**F)** Risk distribution analysis showed that HF samples consistently exhibited significantly higher risk scores than healthy samples in both GSE141910 and GSE57338.

HF biomarkers with diagnostic significance were identified using three machine learning algorithms. The SVM-RFE algorithm identified 12 genes (Figure 3A), the RF model identified 17 genes (Figure 3B). And the LASSO regression analysis yielded 19 genes (Figure 3C). The intersection of these genes using a Venn diagram revealed 10 robust core biomarkers (WT1, CYBB, LPAR2, MTUS2, AURKA, FMN1, TP63, GZMB, CTSK and IL-10) (Figure 3D). The AUC values of the ROC curves shows that AUC of all biomarkers were more than 0.7 (Figure 3E). CYBB, MTUS2, AURKA, FMN1 and IL-10 have a decrease expression in HF, while LPAR2, TP63, GZMB, CTSK, WT1 has a higher expression in HF (Figure 3F). The Rms package was used to construct an HF diagnostic column line graph (Figure 3G). The expression differences of the 10 core CIC-related genes between HF and normal tissues are shown in Figure 3H. Furthermore, DCA revealed that the clinical net benefit of the diagnostic column line graph was higher than that of all other strategies (Figures 4A,B). Additionally, the diagnostic column line graph increased high AUC values in the training cohort (GSE141910), and validation cohort (GSE57338) (0.996, 0.967, respectively; Figures 4C,D). HF samples have a much higher risk score than healthy samples (Figures 4E,F). These results validate the enhanced predictive performance of the diagnostic column line graph.

### Experimental validation of key CIC-related biomarkers in a TAC-induced HF rat model

**FIGURE 5:**
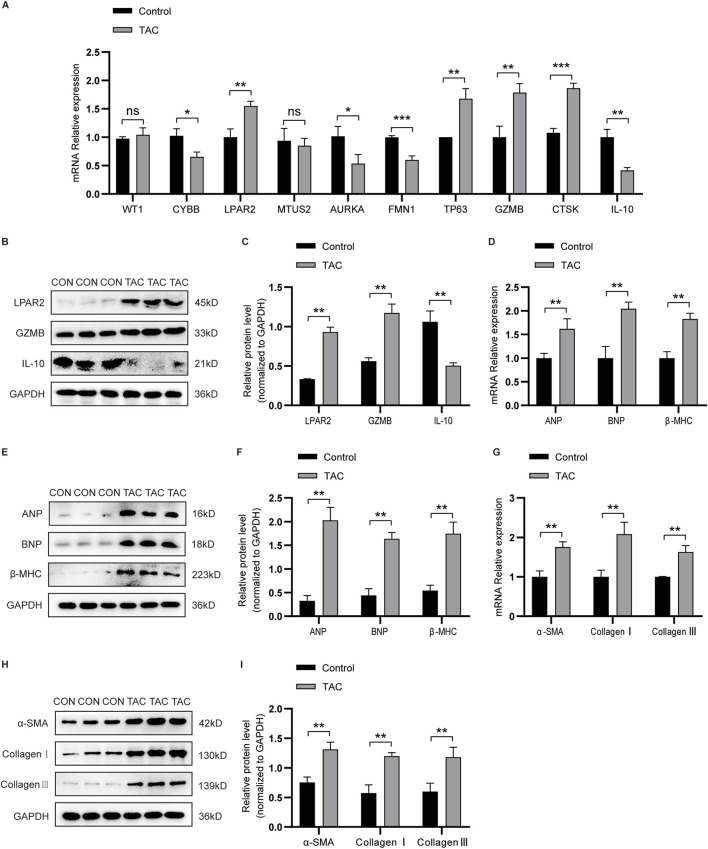
Experimental validation of key CIC-related gene expression in a TAC-induced heart failure model (n = 6 rats per group). **(A)** qRT-PCR showing mRNA levels of 10 core CIC-related genes in sham and TAC hearts. LPAR2 and GZMB were upregulated, while IL-10 was downregulated in TAC rats. **(B,C)** Western blot analysis and quantification of LPAR2, GZMB, and IL-10 protein levels (normalized to GAPDH). **(D)** qRT-PCR of HF markers ANP, BNP, and β-MHC. **(E,F)** Western blot and quantification of ANP, BNP, and β-MHC. **(G)** qRT-PCR of fibrosis markers α-SMA, Collagen I, and Collagen III. **(H,I)** Western blot and quantification of α-SMA, Collagen I, and Collagen III. Protein levels were normalized to GAPDH. qRT-PCR and Western blotting data are shown as the mean ± SEM. Significance: *P < 0.05, **P < 0.01, ***P < 0.001.

To validate these bioinformatics findings, we first performed quantitative qRT-PCR on all 10 core CIC-related genes at the transcriptional level to assess their expression patterns in cardiac tissues from TAC-induced HF rats and corresponding sham controls. As illustrated in Figure 5A, the qRT-PCR results revealed distinct expression trends for the 10 core genes: among them, LPAR2 and GZMB exhibited marked upregulation in TAC-induced HF tissues compared to sham controls, while IL-10 showed a significant downregulation. These transcriptional changes were consistent with the transcriptomic data obtained from human HF samples, confirming the cross-species conservation of CIC-related gene dysregulation in pressure-overload-induced HF. To further corroborate these observations at the protein level and explore the functional relevance of key genes, we selected three representative CIC-related genes—LPAR2, GZMB, and IL-10—for subsequent Western blot analysis. Western blot results (Figures 5B–I) validated the qRT-PCR findings: LPAR2 and GZMB protein levels were significantly elevated in TAC hearts relative to sham controls, whereas IL-10 protein expression was notably reduced. Additionally, we detected increased expression of HF hallmark proteins (ANP, BNP, and β-MHC) and fibrotic markers (α-SMA, Collagen I, and Collagen III) in the TAC model, confirming the successful establishment of the HF phenotype and linking the dysregulation of LPAR2, GZMB, and IL-10 to pathological cardiac remodeling and fibrosis.

### Single-cell transcriptome data analysis

**FIGURE 6:**
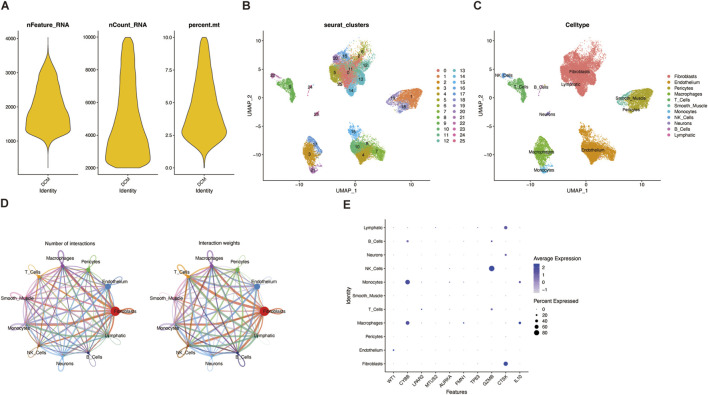
HF related diagnostic biomarkers in the single-cell transcriptome **(A)** Quality control **(B)** A UMAP plot representing the 26 clusters across 20,161 cells from HF samples **(C)** Cell types identified by marker genes **(D)** Interaction analysis of cell types in the HF sample, showing interaction number and strength **(E)** Bubble plot of the average and percent expression of diagnostic biomarkers in different cell subtypes.

We used the HF single-cell dataset GSE183852 to analyze the expression of HF related diagnostic biomarkers in the HF microenvironment. After integrating data from GSE183852 dataset, a total of 20,161 cells were identified. Cells with fewer than 200 total RNAs or more than 2,500 total RNAs were excluded, and cells with more than 5% mitochondria UMI rate were excluded (Figure 6A). After selecting the top 2000 highly variable genes, linear dimensionality reduction was performed to identify the available dimensions of the dataset. First 15 principal components were used to generate the UMAP visualization. There are 11 major cell types in the GSE183852 dataset (Figures 6B,C), including Fibroblasts, Endothelium, Pericytes, Macrophages, T Cells, Smooth Muscle cells, Monocytes cells, NK Cells, Neurons cells, B Cells and Lymphatic cells. The interaction net number and in interaction weight/strength of each celltype were shown in Figure 6D. CellChat analysis further revealed that fibroblasts and endothelial cells served as central hubs in the cellular communication network of the HF microenvironment. The majority of these were Fibroblasts cells and Endothelium cells. WT1 are highly expressed in Endothelium, CYBB are highly expressed in Macrophages and Monocytes, while CTSK are highly expressed in Fibroblasts (Figure 6E).

### Identifying CIC related molecular subgroups and differences in the immune microenvironment between subgroups in HF

**FIGURE 7:**
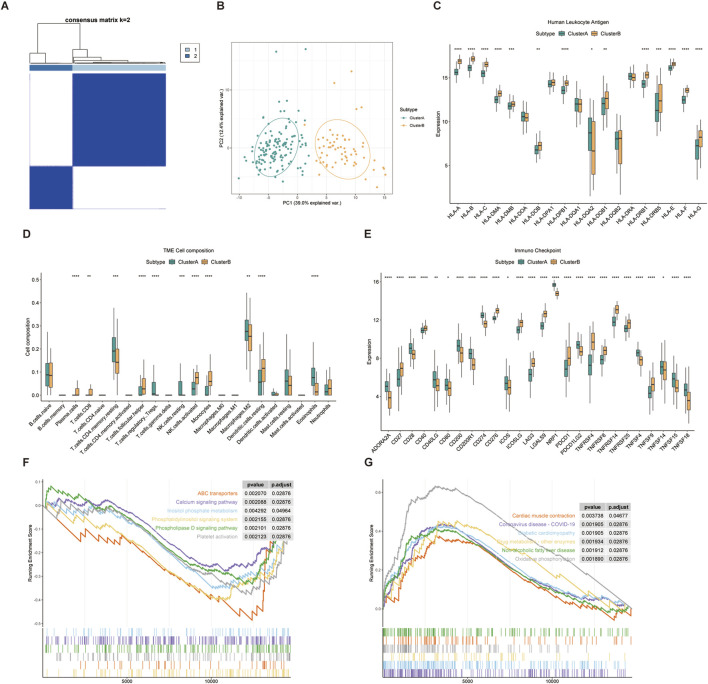
Identification of CIC-related molecular subgroups and immune microenvironment characteristics **(A)** Consensus clustering matrix indicating k = 2 as the optimal cluster number **(B)** PCA plot demonstrating distinct separation between the two subtypes **(C)** Expression profiles of HLA genes between subtypes **(D)** Comparison of immune cell infiltration abundance between subtypes. Cluster A shows higher infiltration of Tregs and M2 macrophages, while Cluster B is enriched in CD8^+^ T cells and NK cells **(E)** Differential expression of immune checkpoint genes **(F,G)** Gene Set Enrichment Analysis (GSEA) revealing distinct pathway activities. Cluster A is associated with immune regulation pathways, whereas Cluster B shows enrichment in metabolic and cardiac contraction pathways.

To explore the connections between the expression of the 101 CIC related genes and HF subtypes, we performed a consensus clustering analysis with all 200 HF patients in the GSE141910 dataset. By increasing the clustering variable (k) from 2 to 10, we found that when k = 2, the intragroup correlations were the highest and the intergroup correlations were low, indicating that the HF patients could be well divided into two clusters based on the above 101 CIC related genes (Figure 7A). 137 cases were included in Cluster A and 63 cases were included in Cluster B. The PCA plot visually demonstrated that there were distinct gene expression patterns between these two clusters, as shown in Figure 7B. Furthermore, Immune infiltration analysis showed that the proportion of 22 types of immune cells significantly different between subgroups (Wilcoxon test, P < 0.05) (Figure 7C). Specifically, Pattern A exhibited higher levels of infiltrated T cells CD4 memory resting, T cells regulatory Tregs, Macrophages M2, and Eosinophils. On the other hand, Pattern B displayed enrichment in T cells CD8, T cells follicular helper, activated NK cells. Regarding immune checkpoints, the expression levels of CD274, ICOS, NRP1, PDCD1LG2, TNFSF4, TNFSF15, and TNFSF18 were notably increased in the ClusterA group, while the expression levels of CD27, CD276, ICOSLG, LAG3, LGALS9, PDCD1, TNFRSF14, TNFRSF25, and TNFSF9 were significantly decreased compared to the ClusterB group (Figure 7D). As shown in Figure 7E, the expression levels of HLA-A, HLA-B, HLA-C, HLA-E, HLA-F and HLA-G was significantly higher in ClusterB than in patients in ClusterA, while HLA-DQA2 was significantly higher in ClusterA than in patients in ClusterB.

To explore the underlying molecular mechanism between CIC related subtypes, we screened out a total of 3,930 subtype related genes (Supplementary Table S2). Gene set enrichment analysis was employed to gain deeper insights into the variations in pathway activity between the two subclusters. The results from GSEA indicated that pathways such as ABC transporters, Inositol phosphate metabolism, Phosphatidylinositol signaling system, and Platelet activation were enriched in the ClusterA group. Conversely, the ClusterB group exhibited enrichment in pathways including Cardiac muscle contraction, Coronavirus disease - COVID-19, Diabetic cardiomyopathy, Drug metabolism - other enzymes, Non-alcoholic fatty liver disease, and Oxidative phosphorylation (Figures 7F,G).

## Discussion

HF remains a severe global health burden with a poor prognosis, highlighting the urgent need to elucidate its pathogenesis beyond the traditional hemodynamic framework (Ceasar et al., 2026; Krishnan et al., 2025; Sun et al., 2026). The intricate interplay between chronic inflammation and adverse cardiac remodeling has placed the immune microenvironment (IME) at the center of HF progression (Huang et al., 2025; Song et al., 2025; Thal et al., 2025). However, the precise cellular mechanisms regulating this complex environment are not yet fully understood.

This study is the first to explore the potential role of CIC- CRGs, a group of genes regulating non-classical cell-cell interaction processes, in shaping the HF immune microenvironment. We acknowledge that adult cardiomyocytes are terminally differentiated; thus, the identified CRGs likely reflect CIC-like interactions among infiltrating immune cells or between immune cells and cardiomyocytes, rather than cardiomyocyte-intrinsic proliferation. By integrating bioinformatics and experimental approaches, we identified a robust 10-CRG diagnostic signature, revealed two HF subtypes with distinct immune landscapes based on CRG expression patterns, and validated the dysregulation of key CRGs in a pressure-overload HF model. Our findings suggest that CRGs are not merely passive biomarkers but may actively participate in regulating the characteristic inflammatory and fibrotic responses in the failing heart, offering new perspectives for understanding HF heterogeneity and developing targeted immunomodulatory strategies.

The 10 core CRGs cross-screened by three machine learning algorithms not only constitute a powerful diagnostic biomarker panel but also reveal the pathophysiological mechanisms of HF from multiple perspectives. Particularly worthy of in-depth discussion are the three genes we selected for experimental validation: LPAR2, GZMB, and IL-10. This selection was based on their significance in differential expression, their high weight in the diagnostic model, and their central roles in known pathways of inflammation, fibrosis, and immune regulation, representing the typical axis of “pro-damage” versus “protective repair” imbalance in the HF immune microenvironment.

First, the upregulation of LPAR2 suggests abnormal activation of the lysophosphatidic acid signaling pathway in HF. LPAR2 is a member of the G protein-coupled receptor family, and its ligand lysophosphatidic acid is a crucial lipid mediator playing a key role in inflammatory responses and tissue fibrosis (Ara et al., 2022; Hutka et al., 2024; Kim et al., 2024). In the cardiovascular field, research has shown that lysophosphatidic acid can activate cardiac fibroblasts, promoting collagen synthesis and secretion, thereby directly driving myocardial interstitial fibrosis (Axelsson Raja et al., 2022; Becker et al., 2020; Schlittler et al., 2023; Sheng et al., 2025). Our finding that LPAR2 is significantly upregulated in both HF patients and TAC rat models is consistent with its potential mediation of sustained inflammatory stimulation and fibrotic responses under pressure overload. Its upregulation may promote the transformation of fibroblasts into myofibroblasts, exacerbating extracellular matrix deposition and thus worsening ventricular stiffness and function (Linna-Kuosmanen et al., 2024; Salminen, 2024; Sun et al., 2022). Mechanistically, LPAR2 primarily signals through Gα12/13 proteins to activate the Rho/Rho-associated coiled-coil containing protein kinase (ROCK) pathway (Wu and Casey, 2024). In cardiac fibroblasts, this signaling cascade promotes stress fiber formation, upregulates the expression of α-smooth muscle actin (α-SMA), and drives the phenotypic transformation of quiescent fibroblasts into active myofibroblasts, which are the primary effectors of extracellular matrix remodeling (Porritt et al., 2026). Importantly, this pro-fibrotic mechanism aligns with our GSEA results, which showed enrichment of pathways related to platelet activation—a process known to release lysophosphatidic acid—in the fibrotic Cluster A subtype. Thus, targeting LPAR2 with specific antagonists represents a promising therapeutic strategy to attenuate cardiac fibroblast activation and halt the progression of adverse fibrotic remodeling in HF.

Second, GZMB is particularly notable for its significant upregulation in HF tissues from both human cohorts and our TAC rat model. GZMB is a serine protease primarily secreted by cytotoxic CD8^+^ T cells and NK cells to induce target cell apoptosis. Its elevated expression in HF aligns with a state of enhanced intramyocardial cytotoxic immune activity (Gao et al., 2025; Ge et al., 2025; Yang et al., 2026). Previous studies have shown that GZMB promotes cardiomyocyte apoptosis and amplifies inflammatory responses in various cardiac pathologies, including myocarditis and ischemia-reperfusion injury (Learmonth et al., 2023; Santos-Zas et al., 2021; Shen et al., 2016). Our single-cell analysis further localized GZMB expression predominantly to T cell and NK cell populations within the HF microenvironment, strengthening its link to cytotoxic lymphocyte infiltration. Given the terminally differentiated nature of cardiomyocytes, this suggests that CRG-mediated processes likely involve immune cell activation and interaction (e.g., emperipolesis-like events) rather than direct cardiomyocyte engulfment, thereby promoting myocardial injury and escalating inflammation in HF. The pro-apoptotic effect of GZMB is mediated through the perforin/granzyme pathway: upon immune synapse formation, perforin facilitates GZMB entry into target cells, where it cleaves and activates caspases, particularly caspase-3, leading to DNA fragmentation and apoptotic cell death (Liu et al., 2024). Beyond its direct cytotoxic effects, GZMB can also cleave extracellular matrix proteins and activate pro-inflammatory cytokines such as IL-1β, thereby amplifying local inflammatory responses and creating a vicious cycle of tissue damage and immune recruitment (Yang et al., 2024). This elevated cytotoxic activity is consistent with our GSEA findings in the immune-activated Cluster B, which showed enrichment of pathways such as “oxidative phosphorylation” (reflecting heightened metabolic demand in activated immune cells) and “COVID-19”a disease characterized by hyperinflammation). Consequently, therapeutic strategies aimed at inhibiting GZMB activity—for instance, with specific serine protease inhibitors—could confer cardioprotection by limiting cytotoxic lymphocyte-mediated myocardial injury and reducing inflammation.

In stark contrast to the pro-inflammatory GZMB, IL-10, a key anti-inflammatory cytokine, is significantly downregulated in HF. IL-10 is crucial for resolving inflammation, promoting tissue repair, and maintaining immune homeostasis by suppressing pro-inflammatory cytokine production and modulating macrophage polarization toward an M2 repair phenotype (Alghamdi et al., 2025; Learmonth et al., 2023; Luo et al., 2025). Its deficiency has been associated with exacerbated cardiac remodeling and worse outcomes in HF models (Ni et al., 2025; Ranjan et al., 2025). Our validation in the TAC model confirmed a significant reduction in IL-10, indicating impaired anti-inflammatory and reparative capacity in the failing heart. Interestingly, despite low IL-10 expression, the CRG-defined subcluster A showed higher infiltration of regulatory Tregs and M2 macrophages, which are classic IL-10 producers (Chen et al., 2025; Shook et al., 2025). This apparent paradox may point to a state of “functional immune exhaustion” or impaired IL-10 signaling within these supposedly anti-inflammatory cells, as observed in chronic inflammatory diseases (Berbers et al., 2021; García-Torre et al., 2024; Hanna et al., 2021). Thus, dysregulation of IL-10 may reflect a critical breakdown of the feedback loops that normally suppress cardiac inflammation. Mechanistically, IL-10 exerts its immunomodulatory effects primarily by binding to the IL-10 receptor complex, leading to activation of the JAK1/STAT3 signaling pathway. This cascade suppresses the expression of pro-inflammatory cytokines (e.g., TNF-α, IL-6, IL-1β) in macrophages and dendritic cells, while simultaneously promoting the polarization of macrophages toward the anti-inflammatory M2 phenotype, which is essential for tissue repair (Singh et al., 2024; 2025; Yu et al., 2024). The downregulation of IL-10 disrupts these protective feedback loops, contributing to the persistent inflammation observed in HF. This interpretation aligns with the GSEA profile of Cluster A, which exhibited enrichment of metabolic pathways (e.g., ABC transporters) but a lack of active inflammatory signaling, suggesting a fibrotic rather than a resolving phenotype. Therefore, restoring IL-10 signaling—through recombinant protein administration or gene therapy—could serve as an effective immunomodulatory strategy to re-establish immune homeostasis and promote tissue repair in HF.

Collectively, these expanded mechanistic insights into LPAR2-driven fibrosis, GZMB-mediated cytotoxicity, and IL-10 deficiency-induced immune dysregulation illustrate how CRGs orchestrate key pathological processes in HF. By linking these molecular events to the specific pathway enrichments from our GSEA—pro-fibrotic pathways in Cluster A and cytotoxic/immune-activated pathways in Cluster B—we provide a more comprehensive understanding of how CIC-related gene networks contribute to HF heterogeneity and progression, reinforcing their potential as both diagnostic biomarkers and therapeutic targets.

Based on the expression patterns of the 101 CRGs, we identified two molecular subtypes of HF with distinct immune microenvironments, offering a new lens through which to examine HF heterogeneity. Subcluster A is characterized by enrichment of resting CD4^+^ memory T cells, Tregs, M2 macrophages, and elevated expression of various immune checkpoint molecules (e.g., CD274/PD-L1, ICOS). This profile resembles an “immunosuppressive and fibro-reparative” phenotype, potentially indicative of advanced HF, where chronic inflammation has transitioned to a state dominated by fibrosis and a compensatory anti-inflammatory response (Buehning et al., 2025; Conte et al., 2025; Tomaszewski et al., 2024). The higher expression of CTSK and TP63 in this subtype supports active fibrotic processes. The upregulation of immune checkpoints might represent an adaptive attempt to limit excessive immune-mediated damage, potentially leading to T cell exhaustion and impaired pathogen/tumor surveillance functions, as observed in cancer (Gao et al., 2025; Nosaka et al., 2025; Ochoa-Espinosa et al., 2025). In contrast, Subcluster B exhibits a microenvironment dominated by CD8^+^ T cells, follicular helper T cells, activated NK cells, and high expression of HLA class I molecules. This aligns with an “immune-activated and cytotoxic” phenotype, which might be more prevalent in early inflammatory stages of HF or in specific etiologies (e.g., viral myocarditis or active autoimmune involvement). The enrichment of pathways like “cardiac muscle contraction” and “oxidative phosphorylation” in Subcluster B, coupled with high expression of GZMB and CYBB, paints a picture of a heart still striving to maintain metabolic function but under direct attack from cytotoxic immune responses. This stratification suggests that CRG expression patterns can distinguish HF patients with fundamentally different underlying immune states, which could have implications for personalized treatment strategies.

Experimental validation in the TAC-induced HF rat model strengthens the translational relevance of our bioinformatics findings. The consistent upregulation of LPAR2 and GZMB and downregulation of IL-10 across species (human HF and murine TAC model) confirms the conservation of these CRG expression changes in pressure-overload HF. This not only validates the robustness of our diagnostic markers but also implies their active involvement in pathophysiological processes, rather than being mere epiphenomena.

Our study holds several promising clinical implications. First, the 10-CRG-based nomogram demonstrates excellent diagnostic accuracy, showing potential as an auxiliary tool for HF diagnosis or risk stratification. More importantly, the identification of two CRG-based immune subtypes paves the way for precision immunomodulation in HF. Certainly, this study also has limitations. First, although we employed a strict training-validation separation to minimize platform heterogeneity, the data were derived from public repositories with limited sample sizes compared to multicenter clinical cohorts. Thus, the diagnostic model lacks validation in a prospective clinical cohort, which we plan to address by collaborating with clinical institutions to validate the 10-gene model against clinical outcomes. Second, while we selected machine learning algorithms for their complementary strengths, we acknowledge inherent limitations like variable selection bias and interpretability issues, though these were minimized through rigorous cross-validation. Third, CIC phenomena were inferred indirectly from gene expression without direct microscopic evidence. Future work will combine transmission electron microscopy and multiplex immunofluorescence to visualize CIC structures in TAC-induced models and human samples, alongside analyzing co-localization with markers like E-cadherin to clarify causal roles. Additionally, we acknowledge that the single-cell dataset used here primarily profiles non-myocyte populations due to dissociation biases against large cardiomyocytes; future single-nucleus sequencing will be needed to profile cardiomyocyte-specific CRG expression.

## Conclusion

In summary, this integrated analysis provides the first comprehensive evidence linking CIC-related genes to immune microenvironment dysregulation in HF. By identifying a high-performance diagnostic signature, revealing clinically significant immune endotype HF subtypes, and validating key findings *in vivo*, we move beyond correlative studies. Our work proposes that the CIC molecular network is a previously unrecognized regulatory layer in HF pathophysiology, potentially orchestrating the interplay between immune responses and tissue remodeling. Future research should focus on elucidating the specific CIC events occurring in the failing heart, investigating the functional consequences of regulating key CRGs in cardiac cells, and ultimately translating these insights into novel diagnostic and therapeutic strategies tailored to the individual patient’s immune landscape.

## Data Availability

The datasets presented in this study can be found in online repositories. The names of the repository/repositories and accession number(s) can be found below: https://www.ncbi.nlm.nih.gov/, GSE141910 https://www.ncbi.nlm.nih.gov/, GSE57338 https://www.ncbi.nlm.nih.gov/, GSE183852.

## References

[B1] Alghamdi M. Dihoum A. Hakami K. Bhattacharjee A. Brown A. J. M. Singh J. (2025). Dapagliflozin reduces epicardial adipose tissue in patients with heart failure and type 2 diabetes. Diabetes Obes. Metab. 27 (12), 7561–7569. 10.1111/dom.70164 41053997 PMC12587262

[B2] Andreadou I. Ghigo A. Nikolaou P. E. Swirski F. K. Thackeray J. T. Heusch G. (2025). Immunometabolism in heart failure. Nat. Rev. Cardiol. 22 (10), 751–772. 10.1038/s41569-025-01165-8 40544171

[B3] Ara H. Subedi U. Sharma P. Bhattarai S. Sharma S. Manikandan S. (2022). Alteration of cellular energy metabolism through LPAR2-axin2 axis in gastric cancer. Biomolecules 12 (12), 1805. 10.3390/biom12121805 36551233 PMC9775664

[B4] Axelsson Raja A. Wakimoto H. DeLaughter D. M. Reichart D. Gorham J. Conner D. A. (2022). Ablation of lysophosphatidic acid receptor 1 attenuates hypertrophic cardiomyopathy in a mouse model. Proc. Natl. Acad. Sci. U. S. A. 119 (28), e2204174119. 10.1073/pnas.2204174119 35787042 PMC9282378

[B5] Becker R. C. Owens A. P. R. Sadayappan S. (2020). Tissue-level inflammation and ventricular remodeling in hypertrophic cardiomyopathy. J. Thromb. Thrombolysis. 49 (2), 177–183. 10.1007/s11239-019-02026-1 31898271 PMC7001758

[B6] Berbers R. M. Drylewicz J. Ellerbroek P. M. van Montfrans J. M. Dalm V. A. S. H. van Hagen P. M. (2021). Targeted proteomics reveals inflammatory pathways that classify immune dysregulation in common variable immunodeficiency. J. Clin. Immunol. 41 (2), 362–373. 10.1007/s10875-020-00908-1 33190167 PMC7858548

[B7] Buehning F. Lerchner T. Vogel J. Hendgen-Cotta U. B. Totzeck M. Rassaf T. (2025). Preclinical models of cardiotoxicity from immune checkpoint inhibitor therapy. Basic. Res. Cardiol. 120 (1), 171–185. 10.1007/s00395-024-01070-0 39039301 PMC11790694

[B8] Ceasar J. N. Yang L. Eberly L. A. Nathan A. S. Roberts E. T. Reina V. J. (2026). Housing cost burden and outcomes among medicaid beneficiaries with heart failure. JAMA Health Forum 7 (1), e255903. 10.1001/jamahealthforum.2025.5903 41481326 PMC12761336

[B9] Chen D. T. Wan Z. J. Sheng X. P. Rao W. Zhan X. H. Gu J. L. (2025). Effects of higenamine on m1/m2 polarization and osteoclast differentiation in rheumatoid arthritis via the THBS-1/TGF-β signaling pathway Cell. Signal. 134, 111905. 10.1016/j.cellsig.2025.111905 40441469

[B10] Conte S. Firoaguer I. Lledo S. Tran T. T. El Yazidi C. Simoncini S. P. (2025). Distinct inflammatory responses of hiPSC-derived endothelial cells and cardiomyocytes to cytokines involved in immune checkpoint inhibitor-associated myocarditis Cells 14 (17), 1397. 10.3390/cells14171397 40940808 PMC12428595

[B11] Cunin P. Bouslama R. Machlus K. R. Martínez-Bonet M. Lee P. Y. Wactor A. (2019). Megakaryocyte emperipolesis mediates membrane transfer from intracytoplasmic neutrophils to platelets. Elife 8. 10.7554/eLife.44031 31042146 PMC6494422

[B12] Davis S. Meltzer P. S. (2007). GEOquery: a bridge between the gene expression omnibus (GEO) and BioConductor. Bioinformatics 23 (14), 1846–1847. 10.1093/bioinformatics/btm254 17496320

[B13] Engebretsen S. Bohlin J. (2019). Statistical predictions with glmnet. Clin. Epigenetics. 11 (1), 123. 10.1186/s13148-019-0730-1 31443682 PMC6708235

[B14] Fais S. Overholtzer M. (2018). Cell-in-cell phenomena, cannibalism, and autophagy: is there a relationship? Cell. Death Dis. 9 (2), 95. 10.1038/s41419-017-0111-7 29367622 PMC5833709

[B15] Flam E. Jang C. Murashige D. Yang Y. Morley M. P. Jung S. (2022). Integrated landscape of cardiac metabolism in end-stage human nonischemic dilated cardiomyopathy. Nat. Cardiovasc Res. 1 (9), 817–829. 10.1038/s44161-022-00117-6 36776621 PMC9910091

[B16] Fragasso G. Stolfo D. Anker M. S. Bayes-Genis A. Chioncel O. Heymans S. (2025). The crosstalk between immune activation and metabolism in heart failure. A scientific statement of the heart failure association of the ESC. Eur. J. Heart Fail. 27 (9), 1700–1719. 10.1002/ejhf.3703 40521614 PMC12502469

[B17] Gao Y. Luo H. Yang B. Song X. Xiong Z. Bahabayi A. (2025). CD55hi MAIT cells with elevated cytokine secretion and activation markers serve as potential diagnostic indicators in sjögren's disease. J. Immunol. 214 (12), 3283–3293. 10.1093/jimmun/vkaf226 40902041

[B18] García-Torre A. Bueno-García E. Moro-García M. A. López-Martínez R. O. Rioseras B. Díaz-Molina B. (2024). IL-10 indirectly modulates functional activity of CD4(+)CD28(null) t-lymphocytes through LFA-3 and HLA class II inhibition. Immunology 173 (2), 296–309. 10.1111/imm.13824 38922883

[B19] Ge M. Wang L. Zheng B. Zhan L. Cui L. Wang H. (2025). Targeting asparagine potentiates anti-PD-l1 immunotherapy in gastric cancer by enhancing CD8(+) t cell anti-tumor response. Front. Immunol. 16, 1626581. 10.3389/fimmu.2025.1626581 41229436 PMC12603620

[B20] Gergely T. S. G. Drobni Z. F. D. Kallikourdis M. Zhu H. Meijers W. C. Neilan T. G. (2024). Immune checkpoints in cardiac physiology and pathology: therapeutic targets for heart failure. Nat. Rev. Cardiol. 21 (7), 443–462. 10.1038/s41569-023-00986-9 38279046

[B21] Halade G. V. Lee D. H. (2022). Inflammation and resolution signaling in cardiac repair and heart failure. EBioMedicine 79, 103992. 10.1016/j.ebiom.2022.103992 35405389 PMC9014358

[B22] Hanna B. S. Llaó-Cid L. Iskar M. Roessner P. M. Klett L. C. Wong J. K. L. (2021). Interleukin-10 receptor signaling promotes the maintenance of a PD-1(int) TCF-1(+) CD8(+) t cell population that sustains anti-tumor immunity. Immunity 54 (12), 2825–2841. 10.1016/j.immuni.2021.11.004 34879221

[B23] Huang Z. Pan L. Xiao Z. Rao F. Yakupu W. Rouzi A. (2025). Kirenol attenuates pressure overload-induced heart failure by enhancing autophagy in macrophages. Int. J. Cardiol. 440, 133681. 10.1016/j.ijcard.2025.133681 40752803

[B24] Humeres C. Shinde A. V. Hanna A. Alex L. Hernández S. C. Li R. (2022). Smad7 effects on TGF-β and ErbB2 restrain myofibroblast activation and protect from postinfarction heart failure. J. Clin. Invest. 132 (3), e146926. 10.1172/JCI146926 34905511 PMC8803336

[B25] Hutka B. Várallyay A. László S. B. Tóth A. S. S. Scheich B. L. Paku S. N. (2024). A dual role of lysophosphatidic acid type 2 receptor (LPAR2) in nonsteroidal anti-inflammatory drug-induced mouse enteropathy. Acta Pharmacol. Sin. 45 (2), 339–353. 10.1038/s41401-023-01175-7 37816857 PMC10789874

[B26] Ichimura S. Misaka T. Ogawara R. Tomita Y. Anzai F. Sato Y. (2024). Neutrophil extracellular traps in myocardial tissue drive cardiac dysfunction and adverse outcomes in patients with heart failure with dilated cardiomyopathy. Circ. Heart Fail. 17 (6), e011057. 10.1161/CIRCHEARTFAILURE.123.011057 38847093

[B27] Jaarsma T. Hill L. Bayes-Genis A. La Rocca H. P. B. Castiello T. Čelutkienė J. (2021). Self-care of heart failure patients: practical management recommendations from the heart failure association of the european society of cardiology. Eur. J. Heart Fail. 23 (1), 157–174. 10.1002/ejhf.2008 32945600 PMC8048442

[B28] Katoh M. Nomura S. Yamada S. Ito M. Hayashi H. Katagiri M. (2024). Vaccine therapy for heart failure targeting the inflammatory cytokine igfbp7. Circulation 150 (5), 374–389. 10.1161/CIRCULATIONAHA.123.064719 38991046

[B29] Kim M. Sur B. Villa T. Yun J. Nah S. Y. Oh S. (2024). Corrigendum to “gintonin regulates inflammation in human IL-1β-stimulated fibroblast-like synoviocytes and carrageenan/kaolin-induced arthritis in rats through LPAR2” [j. Ginseng res. 47 (1) (January 2023) 168]. J. Ginseng Res. 48 (3), 346. 10.1016/j.jgr.2024.03.006 38707643 PMC11068982

[B30] Koenig A. L. Shchukina I. Amrute J. Andhey P. S. Zaitsev K. Lai L. (2022). Single-cell transcriptomics reveals cell-type-specific diversification in human heart failure. Nat. Cardiovasc Res. 1 (3), 263–280. 10.1038/s44161-022-00028-6 35959412 PMC9364913

[B31] Krishnan V. Khan S. S. Mefford M. T. Shah N. S. (2025). Proportional mortality from heart failure and cardiovascular-kidney-metabolic comorbidities among Asian American, native Hawaiian, and other Pacific Islander adults. J. Card. Fail. 10.1016/j.cardfail.2025.12.003 41482119 PMC12860477

[B32] Learmonth M. Corker A. Dasgupta S. DeLeon-Pennell K. Y. (2023). Regulation of cardiac fibroblasts by lymphocytes after a myocardial infarction: playing in the major league Am. J. Physiol.-Heart Circ. Physiol. 325 (3), H553–H561. 10.1152/ajpheart.00250.2023 37450290 PMC10538980

[B33] Li K. Liu P. Han L. Tian J. Zheng Z. Sha M. (2024). Elucidating ferroptosis mechanisms in heart failure through transcriptomics, single-cell sequencing, and experimental validation Cell. Signal. 124, 111416. 10.1016/j.cellsig.2024.111416 39293745

[B34] Linna-Kuosmanen S. Schmauch E. Galani K. Ojanen J. Boix C. A. Örd T. (2024). Transcriptomic and spatial dissection of human *ex vivo* right atrial tissue reveals proinflammatory microvascular changes in ischemic heart disease Cell. Rep. Med. 5 (5), 101556. 10.1016/j.xcrm.2024.101556 38776872 PMC11148807

[B35] Liu X. Yang J. (2023). Cell-in-cell: a potential biomarker of prognosis and a novel mechanism of drug resistance in cancer. Front. Oncol. 13, 1242725. 10.3389/fonc.2023.1242725 37637068 PMC10449025

[B36] Liu Y. Morley M. Brandimarto J. Hannenhalli S. Hu Y. Ashley E. A. (2015). RNA-seq identifies novel myocardial gene expression signatures of heart failure. Genomics 105 (2), 83–89. 10.1016/j.ygeno.2014.12.002 25528681 PMC4684258

[B37] Liu T. Jin D. Le S. B. Chen D. Sebastian M. Riva A. (2024). Machine learning-directed conversion of glioblastoma cells to dendritic cell-like antigen-presenting cells as cancer immunotherapy. Cancer Immunol. Res. 12 (10), 1340–1360. 10.1158/2326-6066.CIR-23-0721 39051633 PMC11491168

[B38] Lu Y. Xia N. Cheng X. (2021). Regulatory t cells in chronic heart failure. Front. Immunol. 12, 732794. 10.3389/fimmu.2021.732794 34630414 PMC8493934

[B39] Luo Q. Zhang Q. Kong Y. Wang S. Wei Q. (2025). Heart failure, inflammation and exercise. Int. J. Biol. Sci. 21 (8), 3324–3350. 10.7150/ijbs.109917 40520009 PMC12160080

[B40] Markousis-Mavrogenis G. Baumhove L. Al-Mubarak A. A. Aboumsallem J. P. Bomer N. Voors A. A. (2024). Immunomodulation and immunopharmacology in heart failure. Nat. Rev. Cardiol. 21 (2), 119–149. 10.1038/s41569-023-00919-6 37709934

[B41] Mihlan M. Wissmann S. Gavrilov A. Kaltenbach L. Britz M. Franke K. (2024). Neutrophil trapping and nexocytosis, mast cell-mediated processes for inflammatory signal relay Cell. 187 (19), 5316–5335. 10.1016/j.cell.2024.07.014 39096902

[B42] Ni W. Ge X. Liu Y. Chen J. Wang L. Chen L. (2025). CD163(+) macrophages attenuate pressure overload-induced left ventricular systolic dysfunction and cardiac mitochondrial dysfunction via interleukin-10 Basic. Res. Cardiol. 120 (4), 727–744. 10.1007/s00395-025-01114-z 40343453

[B43] Nosaka T. Ohtani M. Yamashita J. Murata Y. Akazawa Y. Tanaka T. (2025). PD-l1(+) tumor-associated macrophages induce CD8(+) t cell exhaustion in hepatocellular carcinoma. Neoplasia 69, 101234. 10.1016/j.neo.2025.101234 41027276 PMC12513114

[B44] Ochoa-Espinosa A. Genty L. Xu L. Akin A. Glatz K. Decembrini S. (2025). Coronin 1 deficiency protects from the development of autoimmune myocarditis by reducing CD4+ t cells. Esc. Heart Fail 12 (5), 3524–3536. 10.1002/ehf2.15384 40696745 PMC12450760

[B45] Paulus W. J. Zile M. R. (2021). From systemic inflammation to myocardial fibrosis: the heart failure with preserved ejection fraction paradigm revisited. Circ. Res. 128 (10), 1451–1467. 10.1161/CIRCRESAHA.121.318159 33983831 PMC8351796

[B46] Porritt H. Dixon A. Chalard A. N. S. Ahmad A. M. Taberner A. Lim K. S. (2026). Spatially controlling cardiac fibroblast-to-myofibroblast transition using young's modulus patterned GelMA hydrogels. Acta Biomater. 214, 143–155. 10.1016/j.actbio.2026.02.048 41759758

[B47] Rakisheva A. Farmakis D. Attanasio A. Genis A. B. Cohen-Solal A. Gulati G. (2025). Prevention of cancer therapy-related cardiac dysfunction and heart failure in cancer patients and survivors. A clinical consensus statement of the heart failure association, the european association of preventive cardiology of the ESC, and the ESC council of cardio-oncology. Eur. J. Heart Fail. 27 (11), 2084–2099. 10.1002/ejhf.3753 40679941

[B48] Ranjan P. Goswami S. K. Dutta R. K. Colin K. Pal H. C. Zhang Q. (2025). Hypertrophic heart failure promotes gut dysbiosis and gut leakage in interleukin 10-deficient mice. Am. J. Physiol.-Heart Circ. Physiol. 328 (3), H447–H459. 10.1152/ajpheart.00323.2024 39854049 PMC12235750

[B49] Ritchie M. E. Phipson B. Wu D. Hu Y. Law C. W. Shi W. (2015). Limma powers differential expression analyses for RNA-sequencing and microarray studies. Nucleic. acids. Res. 43 (7), e47. 10.1093/nar/gkv007 25605792 PMC4402510

[B50] Salminen A. (2024). AMPK signaling inhibits the differentiation of myofibroblasts: impact on age-related tissue fibrosis and degeneration. Biogerontology 25 (1), 83–106. 10.1007/s10522-023-10072-9 37917219 PMC10794430

[B51] Santos-Zas I. Lemarié J. Zlatanova I. Cachanado M. Seghezzi J. C. Benamer H. (2021). Cytotoxic CD8(+) t cells promote granzyme b-dependent adverse post-ischemic cardiac remodeling. Nat. Commun. 12 (1), 1483. 10.1038/s41467-021-21737-9 33674611 PMC7935973

[B52] Schiattarella G. G. Alcaide P. Condorelli G. Gillette T. G. Heymans S. Jones E. A. V. (2022). Immunometabolic mechanisms of heart failure with preserved ejection fraction. Nat. Cardiovasc Res. 1 (3), 211–222. 10.1038/s44161-022-00032-w 35755006 PMC9229992

[B53] Schlittler M. Pramstaller P. P. Rossini A. De Bortoli M. (2023). Myocardial fibrosis in hypertrophic cardiomyopathy: a perspective from fibroblasts. Int. J. Mol. Sci. 24 (19), 14845. 10.3390/ijms241914845 37834293 PMC10573356

[B54] Shen Y. Cheng F. Sharma M. Merkulova Y. Raithatha S. A. Parkinson L. G. (2016). Granzyme b deficiency protects against angiotensin II-induced cardiac fibrosis. Am. J. Pathol. 186 (1), 87–100. 10.1016/j.ajpath.2015.09.010 26610869

[B55] Sheng K. Ran Y. Feng X. Wang Y. Zhou S. Guan Y. (2025). PTN secreted by cardiac fibroblasts promotes myocardial fibrosis and inflammation of pressure overload-induced hypertrophic cardiomyopathy through the PTN-SDC4 pathway. Life Sci. 363, 123356. 10.1016/j.lfs.2024.123356 39765325

[B56] Shook P. L. Wang-Heaton H. Casteel J. L. Dalal S. Singh M. Yakubenko V. (2025). Exogenous ubiquitin differentially modulates the phenotype and function of m1 and m2 macrophages. Cells 14 (12), 879. 10.3390/cells14120879 40558506 PMC12190236

[B57] Singh B. Kumari S. Kureel A. K. Saini S. Prakash S. Shah A. (2024). *In-vitro* evidence indicating that IL-10 causes aging-related hypoalbuminemia via JAK1/STAT3 and CEBP-β. Exp. Cell. Res. 443 (1), 114327. 10.1016/j.yexcr.2024.114327 39536933

[B58] Singh B. Saini S. Kumari S. Shah A. Singh K. Chaturvedi C. P. (2025). Leishmania donovani infection-driven high levels of IL-10 causes hypoalbuminemia in human visceral leishmaniasis. Cytokine 193, 156981. 10.1016/j.cyto.2025.156981 40532289

[B59] Slovin S. Carissimo A. Panariello F. Grimaldi A. Bouché V. Gambardella G. (2021). Single-cell RNA sequencing analysis: a step-by-step overview. Methods Mol. Biol. 2284, 343–365. 10.1007/978-1-0716-1307-8_19 33835452

[B60] Song J. Xu R. Zhang H. Xue X. Ruze R. Chen Y. (2023). Cell-in-cell-mediated entosis reveals a progressive mechanism in pancreatic cancer. Gastroenterology 165 (6), 1505–1521. 10.1053/j.gastro.2023.08.035 37657757

[B61] Song Y. J. Zhao X. Y. Wang L. J. Ning T. Chen M. T. Liu P. (2025). Epicardial adipose tissue and heterogeneity parameters combined with inflammatory cells to predict the value of heart failure with preserved ejection fraction patients post myocardial infarction. Cardiovasc. Diabetol. 24 (1), 192. 10.1186/s12933-025-02720-w 40319313 PMC12049797

[B62] Su Y. Huang H. Luo T. Zheng Y. Fan J. Ren H. (2022). Cell-in-cell structure mediates in-cell killing suppressed by CD44. Cell. Discov. 8 (1), 35. 10.1038/s41421-022-00387-1 35436988 PMC9016064

[B63] Sun C. Tian X. Jia Y. Yang M. Li Y. Fernig D. G. (2022). Functions of exogenous FGF signals in regulation of fibroblast to myofibroblast differentiation and extracellular matrix protein expression. Open Biol. 12 (9), 210356. 10.1098/rsob.210356 36102060 PMC9471990

[B64] Sun Z. Zhang X. Zhao Y. Luo Y. Duan X. Lin Z. (2026). Investigating the association between QRS duration change under GDMT and cardiac structure/function alteration in heart failure: insights from a study of 520 patients. Int. J. Cardiol. 447, 134134. 10.1016/j.ijcard.2025.134134 41478450

[B65] Tang X. Wang P. Zhang R. Watanabe I. Chang E. Vinayachandran V. (2022). KLF2 regulates neutrophil activation and thrombosis in cardiac hypertrophy and heart failure progression. J. Clin. Invest. 132 (3), e147191. 10.1172/JCI147191 34793333 PMC8803339

[B66] Thal S. C. Shityakov S. Salvador E. Förster C. Y. (2025). Heart rate variability, microvascular dysfunction, and inflammation: exploring the potential of taVNS in managing heart failure in type 2 diabetes mellitus. Biomolecules 15 (4), 499. 10.3390/biom15040499 40305215 PMC12024555

[B67] Tomaszewski M. Styczeń A. Krysa M. Michalski A. Morawska-Michalska I. Hymos A. (2024). Lymphocyte involvement in the pathology of pulmonary arterial hypertension. Int. J. Mol. Sci. 25 (24), 13455. 10.3390/ijms252413455 39769220 PMC11676877

[B68] Wang M. Niu Z. Qin H. Ruan B. Zheng Y. Ning X. (2020). Mechanical ring interfaces between adherens junction and contractile actomyosin to coordinate entotic cell-in-cell formation. Cell. Rep. 32 (8), 108071. 10.1016/j.celrep.2020.108071 32846129

[B69] Wang R. Zhong H. Wang C. Huang X. Huang A. Du N. (2023a). Tumor malignancy by genetic transfer between cells forming cell-in-cell structures. Cell. Death Dis. 14 (3), 195. 10.1038/s41419-023-05707-1 36914619 PMC10011543

[B70] Wang Y. Zhuang H. Jiang X. H. Zou R. H. Wang H. Y. Fan Z. N. (2023b). Corrigendum: unveiling the key genes, environmental toxins, and drug exposures in modulating the severity of ulcerative colitis: a comprehensive analysis. Front. Immunol. 14, 1323997. 10.3389/fimmu.2023.1323997 38045682 PMC10690403

[B71] Wang Z. Xu H. Chen M. Lu Y. Zheng L. Ma L. (2023c). CCL24/CCR3 axis plays a central role in angiotensin II-induced heart failure by stimulating m2 macrophage polarization and fibroblast activation Cell. Biol. Toxicol. 39 (4), 1413–1431. 10.1007/s10565-022-09767-5 36131165 PMC10425496

[B72] Wilkerson M. D. Hayes D. N. (2010). ConsensusClusterPlus: a class discovery tool with confidence assessments and item tracking Bioinformatics 26 (12), 1572–1573. 10.1093/bioinformatics/btq170 20427518 PMC2881355

[B73] Wu D. Casey P. J. (2024). GPCR-gα13 involvement in mitochondrial function, oxidative stress, and prostate cancer. Int. J. Mol. Sci. 25 (13), 7162. 10.3390/ijms25137162 39000269 PMC11241654

[B74] Yang Z. Z. Liu W. Q. Yu H. D. Yu S. X. Li Y. R. Wang Y. F. (2024). Inhibition of GZMB activity ameliorates cognitive dysfunction by reducing demyelination in diabetic mice. Free. Radic. Biol. Med. 225, 53–62. 10.1016/j.freeradbiomed.2024.09.041 39326683

[B75] Yang P. Bao H. Sun G. Zhang Y. (2026). Single-cell dissection of CD8+ t cell-driven immune dysregulation in type 1 diabetes mellitus: mechanistic links to diabetic foot pathogenesis. Clin. Immunol. 283, 110646. 10.1016/j.clim.2025.110646 41308942

[B76] Yu Z. Zhang Q. Wei S. Zhang Y. Zhou T. Zhang Q. (2024). CD146(+)CAFs promote progression of endometrial cancer by inducing angiogenesis and vasculogenic mimicry via IL-10/JAK1/STAT3 pathway. Cell. Commun. Signal. 22 (1), 170. 10.1186/s12964-024-01550-9 38459564 PMC10921754

[B77] Zhang F. Ye J. Zhu J. Qian W. Wang H. Luo C. (2024). Key cell-in-cell related genes are identified by bioinformatics and experiments in glioblastoma. Cancer Manag. Res. 16, 1109–1130. 10.2147/CMAR.S475513 39253064 PMC11382672

[B78] Zheng M. Hu Y. Liu O. Li S. Wang Y. Li X. (2023). Oxidative stress response biomarkers of ovarian cancer based on single-cell and bulk RNA sequencing Oxidative Med. Cell. Longev. 2023, 1261039. 10.1155/2023/1261039 36743693 PMC9897923

[B79] Zhou G. Soufan O. Ewald J. Hancock R. E. W. Basu N. Xia J. (2019a). NetworkAnalyst 3.0: a visual analytics platform for comprehensive gene expression profiling and meta-analysis Nucleic. acids. Res. 47 (W1), W234–W241. 10.1093/nar/gkz240 30931480 PMC6602507

[B80] Zhou Y. Zhou B. Pache L. Chang M. Khodabakhshi A. H. Tanaseichuk O. (2019b). Metascape provides a biologist-oriented resource for the analysis of systems-level datasets. Nat. Commun. 10 (1), 1523. 10.1038/s41467-019-09234-6 30944313 PMC6447622

[B81] Zhou W. W. Dai C. Liu W. Z. Zhang C. Zhang Y. Yang G. S. (2022). Gentianella acuta improves TAC-induced cardiac remodelling by regulating the notch and PI3k/akt/FOXO1/3 pathways. Biomed. Pharmacother. 154, 113564. 10.1016/j.biopha.2022.113564 35988427

