# Supplementary material for: Integrated analysis of cell-in-cell related genes and immune microenvironment in heart failure
Source: Front Cell Dev Biol. 2026 May 8;14:1806426. doi: 10.3389/fcell.2026.1806426 (PMC13194049; doi:10.3389/fcell.2026.1806426)
Supplement: Supplementary file 1 [file Table1.docx]

Table 1. Primer sequences of selected genes.

| **Name** | **Sequence 5′-3′** |  |
| --- | --- | --- |
| GAPDH | Forward | 5′-CCTCGTCCCGTAGACAAAATG-3′ |
|  | Reverse | 5′-TGAGGTCAATGAAGGGGTCGT-3′ |
| CTSK | Forward | 5′-GCAGCAGAATGGAGGCATTG-3′ |
|  | Reverse | 5′-TTCAGGGCTTTCTCGTTCCC-3′ |
| Wt1 | Forward | 5′-CGCCTTCACCTTGCACTTCTC-3′ |
|  | Reverse | 5′-CGAAAGTGACCGTGCTGTATCC-3′ |
| Cybb | Forward | 5′-TGAGGTGGTGATGTTAGTGGGA-3′ |
|  | Reverse | 5′-GCAAAGTGATTGGCCTGAGATT-3′ |
| Lpar2 | Forward | 5′-CCTATCCAGCCTGCTTGTCTTC-3′ |
|  | Reverse | 5′-ATTTACAGTCCAGACCGTCCAAGA-3′ |
| Mtus2 | Forward | 5′-CCGTATCAGCATCTGGAGGAAG-3′ |
|  | Reverse | 5′-CTCGCTTTGAGGTCTTCGTTCT-3′ |
| Aurka | Forward | 5′-TTGAGTGGCATCGGGGTGAC-3′ |
|  | Reverse | 5′-GGTTACTCAGCCGGGAAGC-3′ |
| Fmn1 | Forward | 5′-TCAGGAAGCCGGAACAGACAA-3′ |
|  | Reverse | 5′-CATCTGTTGCTCGCTTGCTTCT-3′ |
| TP63 | Forward | 5′-CAGAGCGTGCTGGTCCCTTAT-3′ |
|  | Reverse | 5′-GCTGCTTTCTGATGCTGTCTT-3′ |
| Gzmb | Forward | 5′-GCCTTCTTCCTTTCCTAGAGGT-3′ |
|  | Reverse | 5′-CGTATAAGGAAGCCGCCACA-3′ |
| IL-10 | Forward | 5′-CCTGGCTCAGCACTGCTATGT-3′ |
|  | Reverse | 5′-TTGGCAACCCAAGTAACCCTTA-3′ |
| ANP | Forward | 5′-AGCGAGCAGACCGATGAAG-3′ |
|  | Reverse | 5′-AGCCCTCAGTTTGCTTTTCA-3′ |
| BNP | Forward | 5′-TTTGGGCAGAAGATAGACCG-3′ |
|  | Reverse | 5′-AGAAGAGCCGCAGGCAGAG-3′ |
| β-MHC | Forward | 5′-TGGAGCTGATGCACCTGTAG-3′ |
|  | Reverse | 5′-ACTTCGTCTCATTGGGGATG-3′ |
| α-SMA | Forward | 5′-CATCAGGAACCTCGAGAAGC-3′ |
|  | Reverse | 5′-TCGGATACTTCAGGGTCAGG-3′ |
| Collagen I | Forward | 5′-GGAAACCTGATGTATGCTTGAT-3′ |
|  | Reverse | 5′-GACTCCTATGACTTCTGCGTCT-3′ |
| Collagen III | Forward | 5′-TGGATTTCAAGATCAACACTGA-3′ |
|  | Reverse | 5′-TCCGGTTTCCATATTACAGAAC-3′ |
