# Supplementary figures and images for: Integrated analysis of cell-in-cell related genes and immune microenvironment in heart failure
Source: Front Cell Dev Biol. 2026 May 8;14:1806426. doi: 10.3389/fcell.2026.1806426 (PMC13194049; doi:10.3389/fcell.2026.1806426)

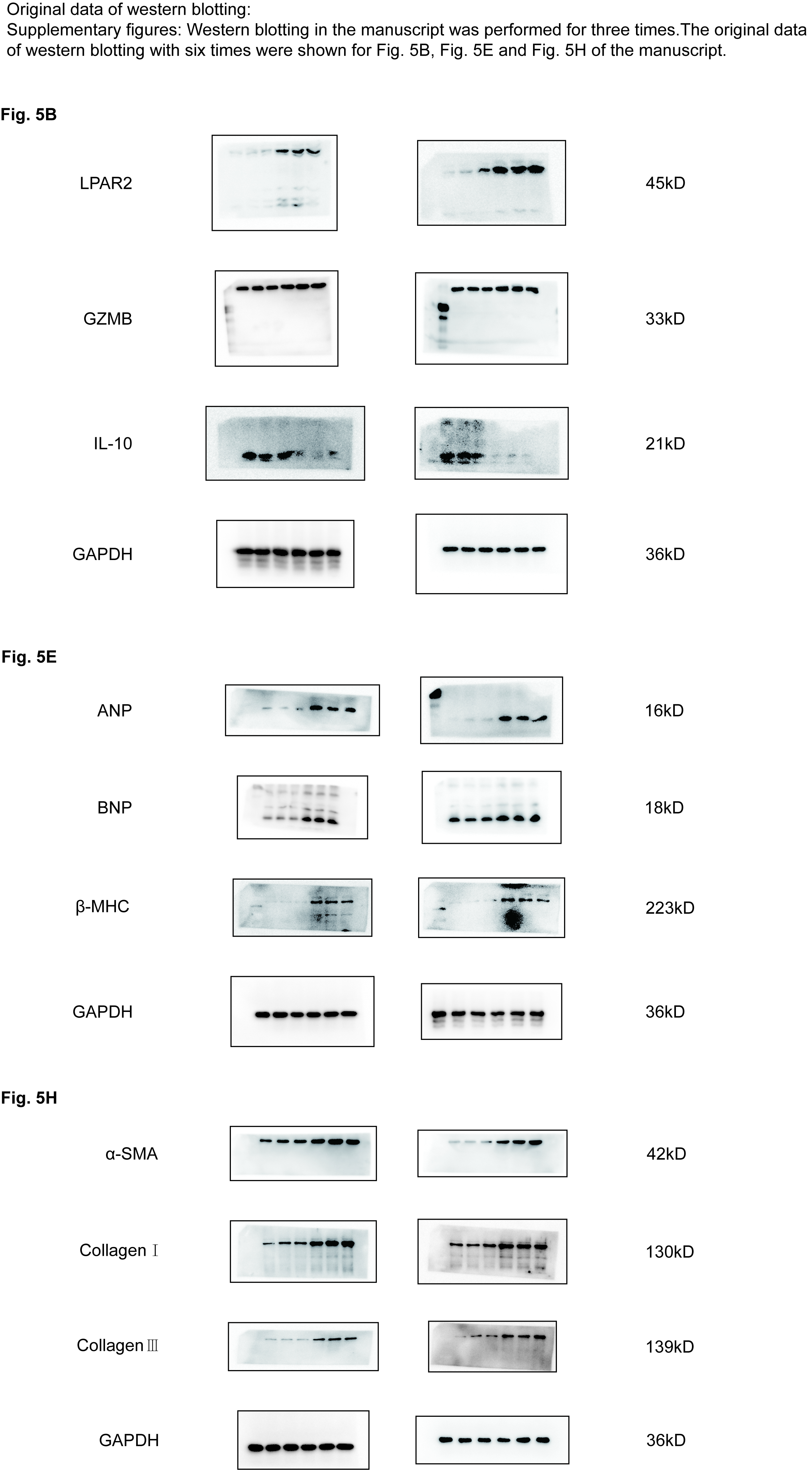

Supplement: Supplementary file 2 [file Image1.tif]
